# An MRI-compatible varus–valgus loading device for whole-knee joint functionality assessment based on compartmental compression: a proof-of-concept study

**DOI:** 10.1007/s10334-020-00844-6

**Published:** 2020-04-20

**Authors:** Oliver Said, Justus Schock, Nils Krämer, Johannes Thüring, Lea Hitpass, Philipp Schad, Christiane Kuhl, Daniel Abrar, Daniel Truhn, Sven Nebelung

**Affiliations:** 1 Department of Diagnostic and Interventional Radiology, Aachen University Hospital, Aachen, Germany; 2 Department of Diagnostic and Interventional Radiology, Medical Faculty, University Hospital Düsseldorf, University Dusseldorf, Moorenstraße 5, 40225 Düsseldorf, Germany; 3 Institute of Computer Vision and Imaging, RWTH University Aachen, Aachen, Germany

**Keywords:** Loading, Stress MRI, Knee joint, Cartilage, Varus

## Abstract

**Objective:**

Beyond static assessment, functional techniques are increasingly applied in magnetic resonance imaging (MRI) studies. Stress MRI techniques bring together MRI and mechanical loading to study knee joint and tissue functionality, yet prototypical axial compressive loading devices are bulky and complex to operate. This study aimed to design and validate an MRI-compatible pressure-controlled varus–valgus loading device that applies loading along the joint line.

**Methods:**

Following the device’s thorough validation, we demonstrated proof of concept by subjecting a structurally intact human cadaveric knee joint to serial imaging in unloaded and loaded configurations, i.e. to varus and valgus loading at 7.5 kPa (= 73.5 N), 15 kPa (= 147.1 N), and 22.5 kPa (= 220.6 N). Following clinical standard (PDw fs) and high-resolution 3D water-selective cartilage (WATSc) sequences, we performed manual segmentations and computations of morphometric cartilage measures. We used CT and radiography (to quantify joint space widths) and histology and biomechanics (to assess tissue quality) as references.

**Results:**

We found (sub)regional decreases in cartilage volume, thickness, and mean joint space widths reflective of areal pressurization of the medial and lateral femorotibial compartments.

**Discussion:**

Once substantiated by larger sample sizes, varus–valgus loading may provide a powerful alternative stress MRI technique.

**Electronic supplementary material:**

The online version of this article (10.1007/s10334-020-00844-6) contains supplementary material, which is available to authorized users.

## Introduction

Magnetic resonance imaging (MRI) is clearly the most powerful and versatile technique for musculoskeletal imaging. Providing the reference standard imaging modality for the non-invasive assessment of intra- and periarticular structures of the human knee joint, current clinical-standard morphological MRI studies are usually performed with the patient in a supine position and their joints in an unloaded and, consequently, unphysiological configuration.

In the past, numerous scanner setups and work-around devices implementing in situ loading have been devised for the sake of more physiological examination conditions. By design, open MRI scanners can examine the patient in a standing or seated position, thereby enabling imaging of the lower extremity under weight-bearing [1, 2]. The undisputed diagnostic benefits of joint imaging in a loaded configuration, however, are realized at the expense of reduced image quality secondary to significantly lower field strengths (i.e. *B*_0_ ≤ 0.5 T), signal-to-noise ratios, and image resolutions [3]. This in turn challenges the reliable detection of slight changes in the intraarticular soft tissues’ morphological appearance [4].

For example, when loaded with half the patient’s body weight, decreases in articular cartilage thickness amount to 3–8% of the initial thickness, equalling absolute deformations of 0.06–0.08 mm [5]. More specifically, the medial femoral condyle undergoes significant relative changes in cartilage thickness of − 3.3 ± 7.1% (normal knees) and − 7.9 ± 11.0% (OA knees) (*p* = 0.097). Relative changes in the medial tibia [− 6.9 ± 14.1% (normal); − 6.2 ± 10.5% (OA); *p* = 0.443] and the lateral tibia [− 4.4 ± 10.3% (normal); − 5.0 ± 8.5% (OA); *p* = 0.452] are less related to degeneration, while changes in the lateral femoral condyle [− 1.0 ± 9.7% (normal); 1.9 ± 8.3% (OA); *p* = 0.243] are considerably less related to loading [5]. Hence, in such functional joint studies, it is pivotal to realize optimized image resolutions and signal-to-noise ratios while maintaining clinically acceptable examination times. As higher field strength may be invested in faster image acquisition, this aspect requires the use of high-field MRI scanners. For these, various prototypical MRI-compatible loading devices for stress MRI in vivo, i.e. for loading a patient’s knee joint(s), have been developed to date [6]. Positioned inside the scanner’s closed horizontal bore along with the patient, these devices provide an alternative way of loading while preserving high imaging standards at feasible examination times. Even though the exact mechanism of loading is different, all these devices apply axial loading along the joint axis, either by suspending weights via dedicated pulley systems [5, 7–10] or by direct compressive loading in a displacement-controlled [11] or pressure-controlled manner [12, 13]. Also, all these devices require mechanical fixation of the patient which renders them bulky, complex, and inconvenient to operate and use. Load application along the leg axis inevitably induces knee flexion (and tibial rotation) [14], which may be partially reduced by immobilization, yet challenges standardization and intra- and inter-patient reproducibility [5, 12]. Despite promising results that indicated the potential of these functional imaging techniques in several experimental and clinical contexts [15], their clinical use is still limited to date.

Against the background of increasing interest in the association of joint mechanics and joint imaging with a traditional focus on cartilage, an alternative approach may be loading of the knee joint along the joint line, i.e. in a varus or valgus configuration, to induce compartmental pressurization of the medial or lateral femorotibial joint compartment. On similar grounds, varus–valgus stress radiography has been applied over the past decades to assess compartmental degeneration in osteoarthritis (OA) in more functional contexts [16–18]. Thereby, quantification of cartilage thickness and tissue laxity was improved as compared to alternative radiographic techniques [17–19], yet clinical adoption was limited due to radiation exposure, impossible direct soft tissue evaluation, and the acquisition of projection radiographs only. Consequently, the fine graduation of early-to-moderate degenerative and non-degenerative cartilage changes by stress radiography remains elusive [17, 20].

Therefore, the present study aimed to bring together varus–valgus loading (VVL) and MRI to improve functional cartilage and joint assessment. This study’s objectives were (1) to design, construct, and validate an MRI-compatible pressure-controlled VVL device for standardized compartmental loading of human knee joints; (2) to demonstrate proof of concept by subjecting a human cadaveric knee joint to a range of VVL intensities and determining MR imaging, computed tomography (CT), and radiographic measures; and (3) to reference these measures to histological and conventional biomechanical measures of sampled joint regions. Consequently, we hypothesized that (1) by means of the VVL device, the medial and lateral femorotibial compartments may be loaded in a standardized, consistent, and reproducible manner and (2) loading-induced adaptive processes within the joint may be reliably detected based on high-resolution morphological MRI techniques, correlated with CT and radiographic measures, and associated with the joint position and loading configuration.

## Materials and methods

### Study design

This study was divided into two parts: (1) the development, construction, and validation of an MRI-compatible pressure-controlled VVL device; and (2) its proof-of-concept application in studying changes within a human cadaveric knee joint based on MR imaging, CT and radiography and as a function of gradually increased VVL intensities.

### Varus–valgus loading (VVL) device

#### System design

**Fig. 1:**
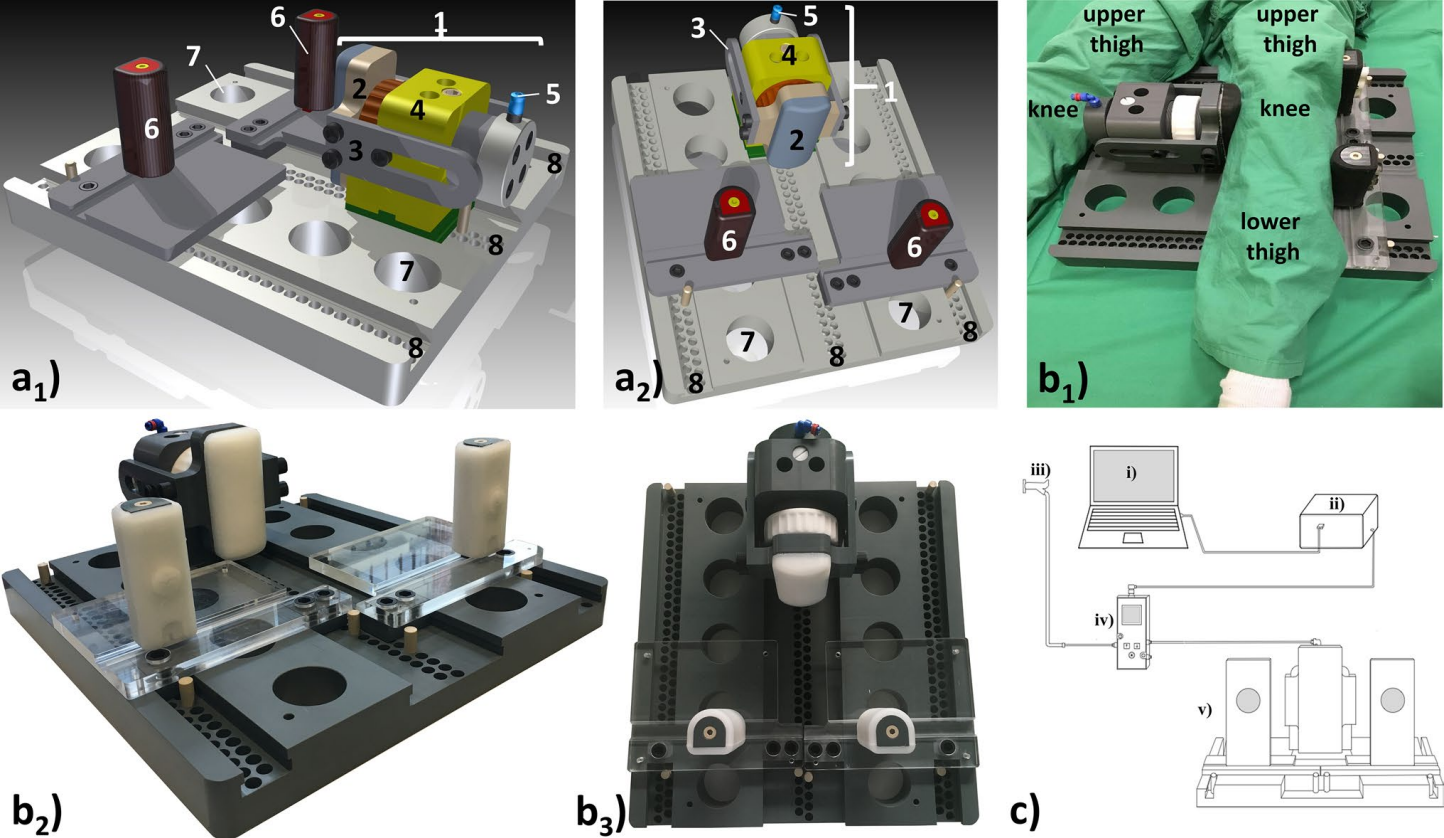
MRI-compatible pressure-controlled varus–valgus loading device [CAD schemes (**a**) and photographs (**b**)] and schematic overview of loading and control unit (**c**). **a** Principally, the pneumatic mechanism (1) consists of the padded pressure applicator (2), safety rails (3) on both sides of the encasement (4) and the loading piston (not shown). The pneumatics are fed remotely via pressure tubes that are connected via pressure port (5). On the opposite side, equally padded counter-bearings (6) to be repositioned in the *X* and *Y* planes ensure reproducible loading of the knee joint with the lower extremity in an anatomical and physiological configuration. The entire setup is mounted on a stable base plate containing openings (7, for weight reduction) and grooves (8, for flexible and symmetrical positioning of the components). **b** Device loaded with a left lower extremity (**b**_**1**_) and unloaded viewed from the side (**b**_**2**_) and from the top (**b**_**3**_). In this setup, the pneumatic mechanism is located at the medial side of the left knee, i.e. in varus loading. **c** Via laptop (i) and conversion device (ii), the pressure level as provided by the hospital’s in-house pressure outlets (iii) is regulated by the pressure control valve (iv) and forwarded to the device (v)

The MRI-compatible pressure-controlled VVL device was laid out as a leverage mechanism, i.e. a central pressure applicator and two opposed counter-bearings as fixed points. Principally, the device consists of a loading and a separate control unit (Fig. 1).

**Fig. 2:**
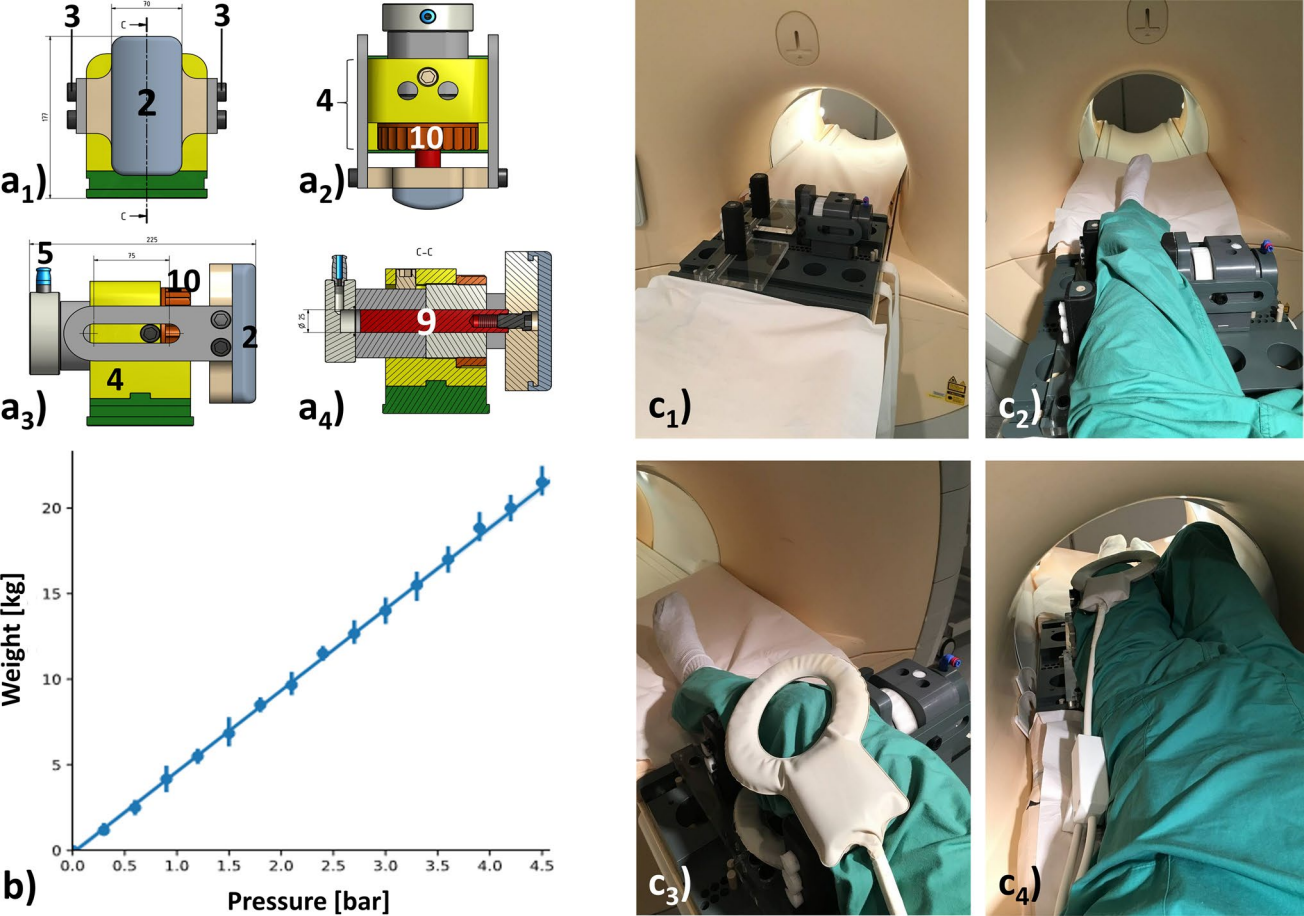
CAD schemes of the pneumatics (**a**), pressure vs. weight calibration curve (**b**), and operation of the MRI-compatible varus–valgus loading device in a 3.0-T MRI scanner (Achieva, Philips) (**c**). **a** Front view (**a**_**1**_), top view (**a**_**2**_), side view (**a**_**3**_), and central cross-section (**a**_**4**_) of the pneumatics. The loading piston (9) has a diameter of 25 mm (= 2.5 cm) and may be adjusted in its horizontal position by means of a coarse set screw (10). Otherwise, component numbering and color coding as in Fig. 1. Units of dimensions are (mm). **b** Theoretical (solid line) and measured (data points) associations between set pressure levels (*x*-axis) and resultant weights or forces (*y*-axis) are plotted. Points give means, while bars give standard deviations (of three measurements). **c** Stepwise loading for eventual MR imaging, i.e. device in the unloaded configuration positioned on the patient bed (**c**_**1**_) and loaded with the senior author’s left lower extremity for varus loading (**c**_**2**_). Multi-purpose measurement coils were used for MR imaging and positioned above and below the knee joint (**c**_**3**_). Final measurement position in the scanner with the contralateral lower extremity placed on the pneumatics (**c**_**4**_)

The loading unit is made of synthetic MRI-compatible materials such as poly(ether-ether-ketone) (PEEK), polymethyl-methacrylate (PMMA), and polyvinyl-chloride (PVC). The solid base plate (PVC, height 5 cm, width 48 cm, length 51 cm) contains full-thickness openings (for weight reduction) and longitudinal grooves (for flexible positioning of the components) and has been dimensioned to safely eliminate any loading-induced bending moments and counter forces. The pneumatic mechanism (Fig. 2a) contains the loading piston and is fed via standard pressure lines (PUN-6X1-BL, Festo, Esslingen, Germany) connected to the pressure port (Festo). Once pressurized (via the associated control unit), the loading piston is actuated and drives out the padded pressure applicator. The pad (thickness: 3 cm, height: 15 cm, width: 7 cm) covering the pressure applicator (PVC, thickness: 2.3 cm, height: 15 cm, width: 7 cm) is made of an equally soft and stable silicone rubber (Dragon Skin^®^ FX-Pro, KauPo Plankenhorn eK, Spaichingen, Germany). This material is fully biocompatible and has been tested for skin irritation (according to DIN EN ISO 10993-10) and cytotoxicity (DIN EN ISO 10993-5). By use of a coarse set screw, the loading piston may be adjusted in its horizontal position, i.e. the *Y* plane, depending on knee joint size. In case of over pressurization, safety rails attached to both sides of the encasement mechanically prevent excessive (and potentially injurious) thrusting of the pressure applicator against the knee joint. On the opposite side of the pneumatic mechanism, counter-bearings ensure reproducible force application by preventing horizontal displacement of both upper and lower thigh. The counter-bearings are semi-circular (PVC, length: 12 cm, width: 3.5 cm, depth: 3.5 cm) and circumferentially surrounded by silicone rubber of 1.0 cm thickness (Dragon Skin^®^ FX-Pro). To account for any interindividual differences in patient anatomy, the counter-bearings are mounted on transparent plates (PMMA, width: 18 cm, length: 24 cm) and may be repositioned in the *X* plane. Correspondingly, the pneumatic mechanism and the two counter-bearings may also be set in various positions along the longitudinal grooves and in the *Y* plane. Fixation of the individual components is realized by locking pins (PEEK) that prevent lateral displacement. The setup may be mirrored for variable VVL configurations of both knee joints.

#### Control unit

The associated control unit is located outside of the MRI scanner room and consists of a digital-to-analogue converter (Multifunction I/O USB-6001, National Instruments, Austin, US) and an electronically actuated pressure valve (VPPM-6L-L-1-G18-0L6H–V1P–S1C1, Festo). Via standard pressure lines these components connect the loading device with the in-hospital pressure outlets that provide pressure levels of up to 4.69 bar. The digital-to-analogue converter is controlled by customized software routines (LabVIEW, National Instruments) and, in turn, controls the pressure valve to any target pressure level from 0 to 4.69 bar. Of note, any compatible air compressor device may be used as alternative pressure source.

Set pressure levels act on a cylindrical pneumatic piston within the device. At the maximum set pressure of 4.69 bar, the piston therefore generates theoretical forces of 230.2 N (pneumatic piston: diameter 25 mm; cross-sectional area: 490.9 mm^2^). As pressure is freely adjustable in the range of 0–4.69 bar, the resultant calculated forces on the pneumatic piston range between 0 and 230.2 N. Force is rigidly transferred to the pressure applicator which is consecutively displaced towards the joint. This force range is reflective of commonly applied force levels for conventional stress radiography of the human knee joint [21].

#### Pressure–force calibration

Mechanical validation of the VVL device and its pneumatic control was performed using a digital hydraulic force gauge [#HKMD29D, Induk, Wuppertal, Germany, specifications: pressure range 0–2.5 kN; accuracy ± 1.0% (of full scale)]. In practical terms, we positioned the load cell between the pressure applicator (without padding) and both counter-bearings that were clamped by a rigid PVC plate. We then determined resultant forces transmitted to the load cell through the pressure applicator for increasing pressure levels set at intervals of 0.3 bar. We repeated these measurements three times and calculated mean forces as a function of set pressure levels. For data analysis, we determined Pearson’s correlation coefficient *r* using the library SciPy in Python (Python Software Foundation, Version 3.6.5) [22] (Fig. 2b). Moreover, we also assessed force levels in a longitudinal manner, i.e. as a function of time. To this end, mean force levels at a constant pressure level of 3 bar were measured every minute over a period of 30 min.

### Human cadaveric knee joint

One fresh and structurally intact left human cadaveric knee joint from a male body donor, who had deceased due to unrelated medical conditions at the age of 78 years, was obtained from the Department of Anatomy (RWTH Aachen University, Germany). Of note, local Institutional Review Board approval (Ethical Committee, RWTH Aachen University, AZ-EK180/16) and written informed consent by the body donor were available at study initiation.

In preparation of the MRI measurements, the human cadaveric knee joint was positioned in slight flexion of approximately 20° by placing appropriate positioning aids underneath the joint. Additionally, we used mechanical positioners such as sandbags to reduce joint motion in response to loading. In varus or valgus loading, the medial or lateral joint line was brought in line with the centre of the padded pressure applicator. Both counter-bearings were adjusted to the anatomy of the knee joint at the greatest possible distance from each other. Attention was paid that the loading components were in loose contact with the knee joint, i.e. the counter-bearings with the lower and upper thirds of the femur and tibia and the pressure applicator with the medial or the lateral joint line.

### Imaging studies

#### MRI studies

The VVL device was loaded with the human cadaveric knee joint and centrally positioned in the bore of a clinical 3.0-T scanner (Achieva, Philips, The Netherlands) (Fig. 2c). Imaging was performed using a general-purpose dual-coil configuration (Sense-Flex L, Philips) with the coils being placed above and below the joint where they were attached with medical adhesive tape to prevent displacement during loading. The presence of the loading components made the use of a dedicated multi-channel knee coil impossible due to space constraints. In preparation of the actual MRI measurements, the presence of image artefacts was assessed using dedicated *B*_0_ and *B*_1_ mapping sequences (as provided by the manufacturer, Philips) acquired with a water phantom with and without the loading device in position. Moreover, we used a T2*-weighted 2D gradient-echo sequence (time to repetition = 702.5 ms, echo time = 16.1 ms, flip angle = 18°, 22 slices, slice thickness = 5 mm, slice gap = 15.5 mm, field of view = 467 mm × 467 mm, acquisition matrix = 256 × 205, number of signal averages = 1; Q-body coil; coronal orientation) to assess the presence of image artefacts because of the loading device. Once the connections and system operability were checked, we performed the MRI measurements in seven distinct configurations: (1) unloaded (*δ*_0_), (2) valgus load of 7.5 kPa (= 73.5 N, *δ*_vlg1_), (3) valgus load of 15 kPa (= 147.1 N, *δ*_vlg2_), (4) valgus load of 22.5 kPa (= 220.6 N, *δ*_vlg3_), (5) varus load of 7.5 kPa (*δ*_var1_), (6) varus load of 15 kPa (*δ*_var2_), and (7) varus load of 22.5 kPa (*δ*_var3_). Of note, pressure levels were set by digital control. Throughout the study, equilibration periods of 5 min were observed after each change in pressure level. For each loading position, fat-saturated 2D proton density-weighted (PD-fs) sequences and 3D water-selective cartilage scans (WATSc) were acquired. Please see Table 1 for details of the chosen sequence parameters. The PD-fs sequences are frequently used for imaging of the knee joint and articular cartilage [23, 24] and were acquired in the sagittal, axial and coronal planes (*δ*_0_) and the axial and coronal planes (*δ*_vlg1_, *δ*_vlg2_, *δ*_vlg3_, *δ*_var1_, *δ*_var2_, and *δ*_var3_), respectively. The WATSc sequence was acquired in the coronal plane and used to perform cartilage segmentations and morphometric measurements [25]. Based on the scout views and the PD-fs sequences, we checked the appropriate joint position within the device and measurements were performed in the seven joint configurations in the order 1–7 as detailed above. The measurements were performed at room temperature. Total magnet time was approximately 3.5 h.

**Table 1:** Acquisition parameters of MR sequences

	PDW fs	WATSc
Orientation	cor, ax (sag)	Cor
Type of fat saturation	SPAIR	Water-selective excitation^a^
Sequence type	Turbo spin echo	Gradient echo
Repetition time (ms)	4776	10
Echo time (ms)	30	5
Turbo spin-echo factor	13	1
Field of view (mm)	180 × 180	180 × 180
Acquisition matrix	368 × 364	368 × 368
Reconstruction matrix	512 × 512	512 × 512
Scan percentage (%)	100	78.5
Flip angle (°)	90	17
Number of signal averages	1	1
Slices	33	266
Slice thickness/gap (mm)	3.0/3.5	1.5/0.0^b^
Duration (min)	5 min 34 s–6 min 39 s	9 min 30 s

Of note, the PDW-fs sequences were obtained in the cor, ax, and sag orientations in the unloaded reference configuration, while in all other configurations, they were only obtained in the cor and ax orientations

*PDW* proton density-weighted, *WATSc* water-selective cartilage scan, *SPAIR* spectral attenuated inversion recovery, *fs* fat-saturated, *n/a* not applicable, *cor* coronal, *ax* axial, *sag* sagittal

^a^Because of its robustness towards *B*_0_ magnetic field inhomogeneities, the binomial scheme 1331 was used for water-selective excitation

^b^A slice increment of 0.75 mm was present, i.e. the centres of two consecutive slices were 0.75 mm apart

#### CT studies

Analogously, the human cadaveric knee joint was scanned sequentially in the craniocaudal direction using a multidetector-row clinical CT scanner (SOMATOM Force, Siemens, Erlangen, Germany) and the following scan parameters: tube voltage 120 kV, tube current 800 mAs, slice thickness 0.6 mm, rotation time 1 sec, increment 3 mm, pitch 0.8. After adapting the field of view (158 mm × 158 mm) to the joint outline, the chosen matrix (512 × 512) resulted in a pixel size of 0.31 mm × 0.31 mm. Scan duration was 5.28 s per joint configuration. For image reconstructions, the Siemens kernel Br64s was used and axial, coronal and sagittal reconstructions were obtained. As for MR imaging, the CT measurements were carried out in all joint configurations (*δ*_0_, *δ*_vlg1_, *δ*_vlg2_, *δ*_vlg3_, *δ*_var1_, *δ*_var2_, and *δ*_var3_) using the pressure-controlled VVL device. An equilibration period of 5 min was observed after each change in pressure.

#### Radiographic studies

Stress radiography was performed using the commercially available Telos GA-III/E device (Telos GmbH) at 20° of knee flexion. Using standard settings for clinical radiography of the knee (60 kV, 4 mAs, variable focus-to-detector distance), anteroposterior projection radiographs of the knee were obtained in the following configurations: *δ*_0_, *δ*_vlg1_, *δ*_vlg2_, *δ*_var1_, and *δ*_var2_. Loading to 22.5 kPa (i.e. *δ*_vlg3_, *δ*_var3_) is not possible with this device.

### Post-processing and image analysis

#### MRI studies

MR images of the human cadaveric knee joint were processed separately and in each configuration. Morphometric measurements of the medial and lateral femorotibial cartilage were based on manual segmentations and computations using Chondrometrics software (Chondrometrics GmbH, Ainring, Germany) as published before, e.g. [26, 27]. On the basis of the 3D WATS-c sequences, the subchondral bone plates and cartilage surfaces of the medial and lateral femorotibial compartments were segmented manually in each image. Readers were blinded to the joint’s configuration and reading quality was checked by an expert reader. Mean cartilage thickness over the subchondral bone was computed based on the segmented bone interface and cartilage surface by generation of 3D reconstructed cartilage surface areas. Subsequently, cartilage morphometric measures were quantified for each loading position. The mean cartilage thickness (ThC) was determined in the medial and lateral femorotibial compartments (MFTC, LFTC), the medial and lateral tibia (MT, LT), the medial and lateral central (i.e. weight-bearing) femur (cMF, cLF), five tibial subregions (central, external, internal, anterior, and posterior) of MT and LT, and three femoral subregions (central, external, and internal) of cMF and cLF. Moreover, cartilage volumes (VC) and cartilage surface areas (AC) were computed based on the manual segmentations and determined for the MT, LT, cMF, and cLF.

#### CT and radiographic studies

For CT analyses, sagittal and axial reconstructions were used to identify the coronal reference plane as the coronal imaging slice that centrally bisected the weight-bearing region of the MFTC and LFTC at the centre of the anteroposterior distance between the deepest point of the trochlear groove and the posterior condylar line. To this end, the joint space widths, i.e. the vertical distances between the subchondral bone contours of the tibial and femoral condyles, were measured at three locations per compartment, i.e. at the inner, central, and outer third. Similarly, joint space widths were measured at three locations on the projection radiographs. Mean joint space width was calculated for each loading position, compartment, and modality.

In addition, potential positional changes of the knee joint in response to loading were determined in terms of joint flexion and rotation. Flexion angle was determined between the femoral and tibial shafts on sagittal reconstructions. Rotation was measured as the angle between the device’s horizontal base plate and a line that joined both corners of the patella on axial reconstructions. One reader (OS, 2 years of experience in MSK radiology) who was blinded to the joint configuration performed these measurements for each joint configuration using the inbuilt calliper tool of the clinical-routine picture archiving and communications system (PACS, iSite, Philips, The Netherlands). For visualization purposes, shaded CT volume renderings were generated using IntelliSpace Clinical Applications (Philips). Because of variable flexion and rotation at the different loading configurations, the knee joint (as the volume-of-interest) was defined and subsequently rolled and rotated to allow for unobstructed visualization of both joint spaces.

### Reference evaluation

Immediately after completion of imaging studies, femorotibial pressure levels as a function of gradually increasing VVL were spatially mapped in the human cadaveric knee joint using digital electronic pressure-sensitive sensors (K-Scan 4000, 1.500 psi, Tekscan, Boston, MA, US). This system has been used widely in static and dynamic pressure mappings of the human knee joint [28, 29]. Its pressure-sensitive area measures 33 mm × 27.9 mm (height × width) and contains 62 sensor elements/cm^2^ to map maximum pressures of up to 103.42 bar (= 10342 kPa). Due to its flexible and thin configuration (thickness: 0.1 mm), the area conforms to the individual knee joint anatomy. After accessing the joint via a medial parapatellar approach, the sensors were inserted into the medial and lateral compartments covering the femoral articular surfaces. Care was taken to avoid crinkling of the sensor areas and to cover the medial- and lateral-most portions of the compartments. However, due to limitations in sensor area, only the anterior two-thirds of the tibiofemoral articular surface were covered. To gain access to the joint, the collateral and cruciate ligament complex had to be partially cut. Prior to the measurements, sensors were preconditioned and calibrated in line with the manufacturer’s instructions. Then, the knee joint was placed into the VVL device and loaded in six configurations, while equilibration periods of 5 min were observed after setting each loading position: 7.5 kPa varus (*δ*_1var_), 15 kPa varus (*δ*_2var_), 22.5 kPa varus (*δ*_3var_), 7.5 kPa valgus (*δ*_1vlg_), 15 kPa valgus (*δ*_2vlg_), and 22.5 kPa valgus (*δ*_3vlg_). Reasoning behind the choice of these loading intensities involves the most commonly used device for stress radiography, i.e. the Telos device (Telos GmbH, current version GA-III/E, Wölfersheim-Berstadt, Germany), which uses 15 kPa (= 147.2 N). The raw data obtained from these measurements were imported into Matlab (MatlabR2018b, Natick, USA) and analysed using customized analysis routines.

Next, the central weight-bearing femoral and tibial joint surfaces were identified and the peripheral femoral and tibial cartilage areas as well as the meniscus body regions were sampled to undergo histological and biomechanical reference assessment as published before [30–34] (see Supplementary File 1 for a detailed description).

## Results

Validation of the pressure–force relationship revealed a close-to-perfectly linear association (Pearson’s *r* = 0.999, *p* < 0.001) between set pressure levels and resultant weights or forces (Fig. 2b). Consequently, set pressures of 1.62 bar, 3.23 bar, and 4.84 bar brought about weights (or forces) of 7.5 kg (= 73.6 N), 15 kg (= 147.2 N) and 22.5 kg (225 N), respectively. Longitudinal assessment of resultant forces at the set pressure level of 3 bar revealed constant measured forces of 130 N and, hence, no loss in force over 30 min. Reference pressure mappings using pressure-sensitive sensors resulted in total forces over the entire loaded sensor area of 14.7 N (*δ*_vlg1_), 46.6 N (*δ*_vlg2_), 50.3 N (*δ*_vlg3_), 13.1 N (*δ*_var1_), 37.7 N (*δ*_var2_), and 38.6 N (*δ*_var3_).

No significant magnetic field inhomogeneity or image artefacts were associated with the presence of the loading device inside the scanner as indicated by *B*_0_ or *B*_1_ mapping and the T2*-weighted sequence, respectively. Overall, handling of the device, positioning of the human cadaveric knee joint and pressure-controlled application of force during MRI and CT studies was realized without difficulties. We did not encounter any adverse or unexpected events. Consequently, the knee joint underwent the MRI, CT and radiographic studies as planned in all configurations.

**Fig. 3:**
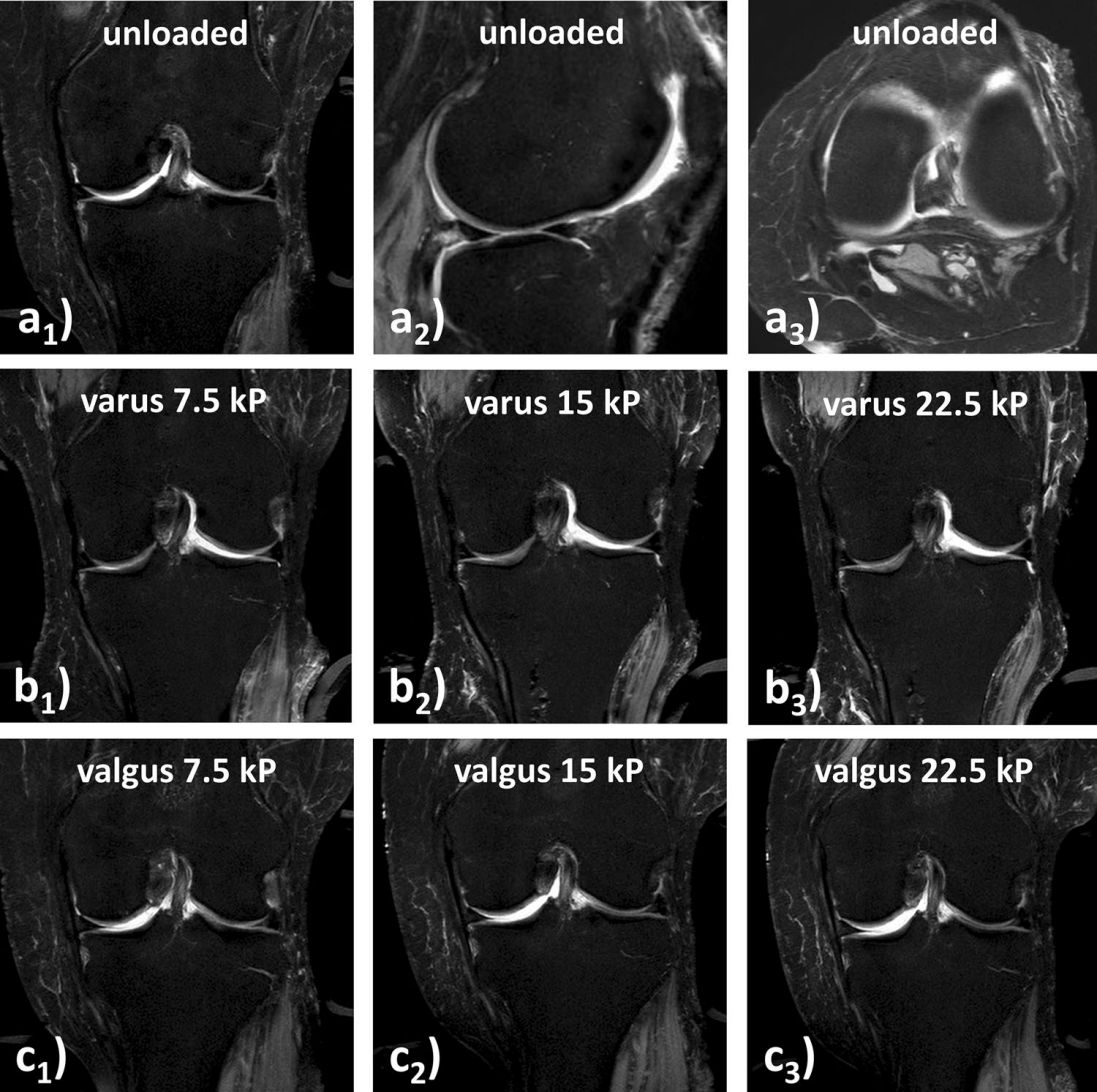
MR imaging of gradually increasing varus and valgus loading of the human cadaveric knee joint. **a** MR images in the unloaded configuration (PDW fs) obtained at three orientations, i.e. coronal (**a**_**1**_, through central weight-bearing region of the femur), sagittal (**a**_**2**_, through lateral femorotibial joint), and axial (**a**_**3**_, through distal femoral condyles). Structurally, the joint was grossly intact. **b** MR images of the coronal reference plane (as in **a**_**1**_) in response to increasing varus loading of 7.5 kPa (**b**_**1**_), 15 kPa (**b**_**2**_), and 22.5 kPa (**b**_**3**_). Medially, progressive narrowing of the femorotibial articulation is accompanied by decreasing amounts of interarticular joint fluid; laterally, progressive distention of the femorotibial articulation. **c** MR images of the coronal reference plane (as in **a**_**1**_ and **b** in response to increasing valgus loading of 7.5 kPa (**c**_**1**_), 15 kPa (**c**_**2**_), and 22.5 kPa (**c**_**3**_). While the lateral femorotibial articulation is progressively compressed, the medial femorotibial articulation is gradually distended. Note the silhouettes of the pressure applicator on the medial (**b**) or lateral (**c**) aspect of the joint and of the counter-bearings on the lateral (**b**) and medial (**c**) aspects

For MRI studies, qualitative evaluation revealed that the medial femorotibial articulation gradually narrowed in size with increasing varus loading intensity, while the lateral femorotibial articulation was gradually distended (Fig. 3a, b). Corresponding changes in both compartments were observed in response to increasing valgus loading intensity (Fig. 3a, c).

Quantitative evaluation of cartilage morphometric measures, i.e. VC and ThC, were reflective of these changes throughout the various loading configurations (Table 2). In the MFTC, VC and ThC decreased in response to increasing varus loading, while these parameters remained largely unchanged (or increased slightly) in response to increasing valgus loading, e.g. mean ThC (cMF): 1.727 mm (*δ*_0_); 1.558 mm (*δ*_var3_); 1.724 mm (*δ*_vlg3_). Corresponding findings were made for the LFTC, where ThC of the lateral femur was slightly decreased in response to valgus loading and increased in response to varus loading, e.g. mean ThC (cLF): 1.637 mm (*δ*_0_); 1.735 mm (*δ*_var3_); 1.591 mm (*δ*_vlg2_); 1.622 mm (*δ*_vlg3_). Generally, changes in VC and ThC of the LFTC in response to valgus loading were less clearly associated with loading intensity than the corresponding changes in the MFTC in response to varus loading.

**Table 2:** Articular cartilage morphometric measures of the medial and lateral femorotibial joint compartments of a human cadaveric knee joint as a function of increasing varus–valgus loading intensity, i.e. unloaded and after 7.5 kPa, 15 kPa, and 22.5 kPa varus–valgus loading

	Unloaded	7.5 kPa varus	15 kPa varus	22.5 kPa varus	7.5 kPa valgus	15 kPa valgus	22.5 kPa valgus
Medial compartment							
Tibia							
VC (mm^3^)	1608.8	1534.7 (− 4.6)	1507.2 (− 6.3)	1500.0 (− 6.8)	1546.2 (− 3.9)	1592.2 (− 1.0)	1614.8 (0.4)
ThC (mm)	1.509 ± 0.604	1.458 ± 0.564 (− 3.4)	1.423 ± 0.553 (− 5.7)	1.416 ± 0.535 (− 6.2)	1.475 ± 0.586 (− 2.3)	1.492 ± 0.586 (− 1.1)	1.514 ± 0.587 (0.3)
AC (cm^2^)	10.86	10.70 (− 1.5)	10.77 (− 0.8)	10.79 (− 0.6)	10.68 (− 1.7)	10.86 (0)	10.88 (0.2)
Femur							
VC (mm^3^)	921.5	868.8 (− 5.7)	827.0 (− 10.3)	819.8 (− 11.0)	941.4 (2.2)	893.6 (− 3.0)	917.1 (− 0.5)
ThC (mm)	1.727 ± 0.523	1.631 ± 0.507 (− 5.6)	1.566 ± 0.491 (− 9.3)	1.558 ± 0.498 (− 9.8)	1.744 ± 0.533 (1.0)	1.674 ± 0.514 (− 3.1)	1.724 ± 0.537 (− 0.2)
AC (cm^2^)	5.65	5.62 (− 0.5)	5.55 (− 1.8)	5.55 (− 1.8)	5.71 (1.1)	5.62 (− 0.5)	5.63 (− 0.4)
Lateral compartment							
Tibia							
VC (mm^3^)	1547.3	1496.8 (− 3.3)	1502.6 (− 2.9)	1517.2 (− 1.9)	1482.7 (− 4.2)	1445.4 (− 6.6)	1471.3 (− 4.9)
ThC (mm)	1.760 ± 0.841	1.712 ± 0.813 (− 2.7)	1.696 ± 0.810 (− 3.6)	1.717 ± 0.854 (− 2.4)	1.680 ± 0.791 (− 4.5)	1.644 ± 0.777 (− 6.6)	1.646 ± 0.753 (− 6.5)
AC (cm^2^)	9.04	8.95 (− 1.0)	9.11 (0.8)	9.05 (0.1)	9.09 (0.6)	8.99 (− 0.6)	9.18 (1.5)
Femur							
VC (mm^3^)	1013.1	1083.8 (7.0)	1089.0 (7.5)	1079.8 (6.6)	1052.8 (3.9)	986.2 (− 2.7)	1009.2 (− 0.4)
ThC (mm)	1.637 ± 0.545	1.750 ± 0.549 (6.9)	1.744 ± 0.553 (6.5)	1.735 ± 0.557 (6.0)	1.697 ± 0.558 (3.7)	1.591 ± 0.533 (− 2.8)	1.622 ± 0.556 (− 0.9)
AC (cm^2^)	6.52	6.55 (0.5)	6.61 (1.4)	6.58 (0.9)	6.56 (0.6)	6.55 (0.5)	6.58 (0.9)

Data are presented as mean value (VC and AC) ± standard deviation (ThC) and [percentage change versus the unloaded configuration (%)]. Morphometric measures were derived from manually segmented high-resolution WATSc sequences

*VC* cartilage volume (mm^3^), *ThC* cartilage thickness (mm), *AC* cartilage surface area (cm^2^)

Increasing the loading intensity in the high-loading range, i.e. 15–22.5 kPa, produced only smaller—and partially inconsistent—changes in VC and ThC as compared to the low-loading range, i.e. unloaded to 15 kPa. Changes in AC were inconsistent and not clearly related to type and intensity of loading.

**Fig. 4:**
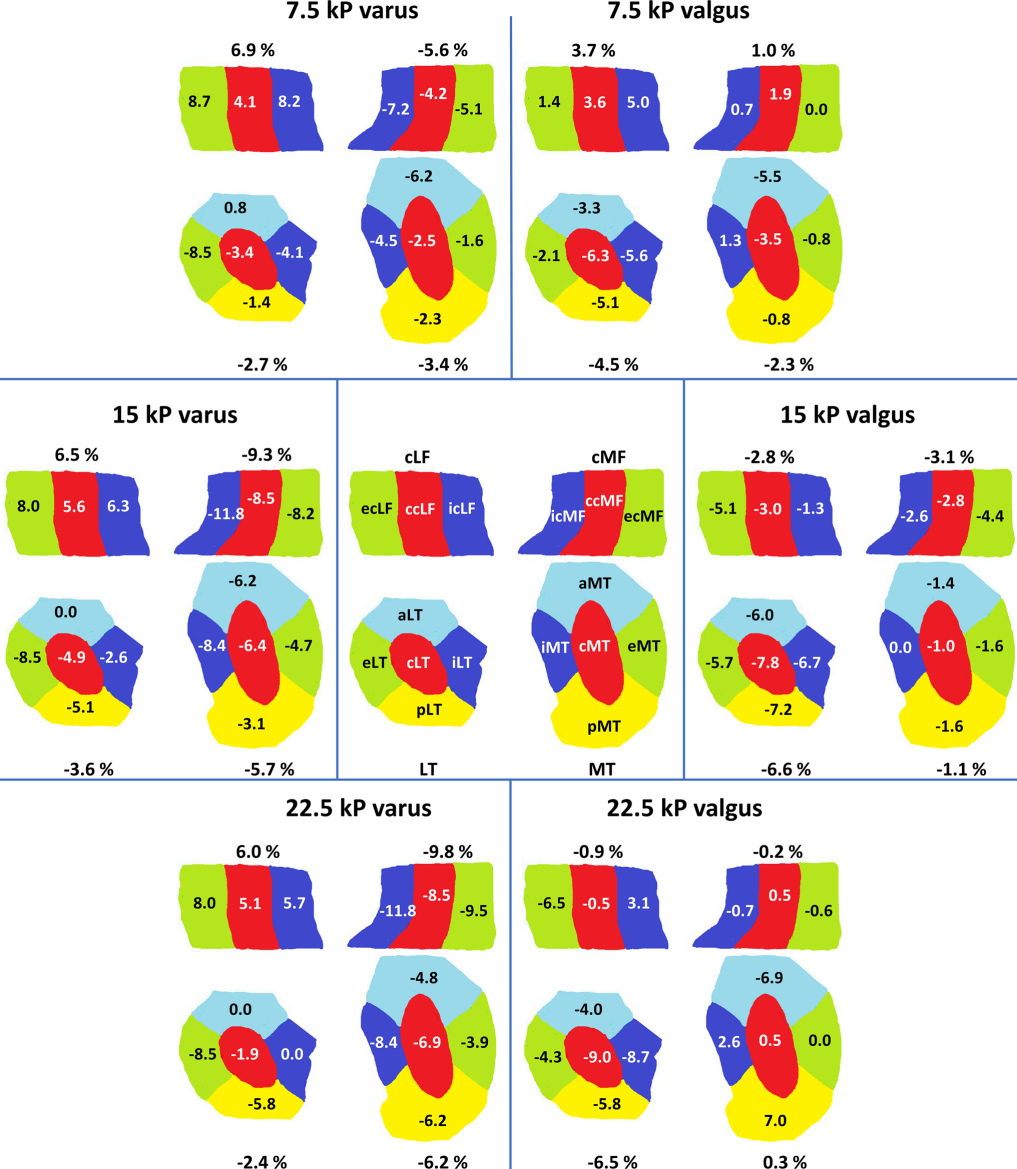
Projections to demonstrate relative changes in ThC in the distinct femoral and tibial regions and subregions as a function of joint configuration, i.e. loading intensity (at 7.5 kPa, 15 kPa, and 22.5 kPa) and direction (varus and valgus). The central part of the medial (cMF) and lateral femur (cLF) were partitioned into the central (c), external (e), and internal subregions (i), while the medial (MT) and lateral tibia (LT) were partitioned into the central (c), external (e), internal (i), anterior (a), and posterior subregions (p). Relative changes in ThC and in the cartilage plate of the region and subregion as compared to the unloaded reference configuration (%). Visualization inspired by [35]

Overall, analysis of the distinct subregions confirmed the above-mentioned findings (Table 3, Fig. 4). In response to varus loading, largest decreases in ThC of cMF and MT were determined in the central and internal subregions, while in the remaining subregions [i.e. external, anterior, and posterior (tibial); external (femoral)] changes were consistent and less pronounced. Correspondingly, largest decreases in ThC in response to valgus loading were found in the central and internal subregions of LT, while for cLF, changes were less consistent. Loading-related decreases in cLF were only found in the external subregion. Closer evaluation of the tibial changes along the anteroposterior dimension revealed that decreases in ThC tended to be larger in anterior than posterior subregions of MT and LT, in particular at higher loading intensities.

**Table 3:** Mean cartilage thickness of the medial and lateral femorotibial joint compartment subregions

	Unloaded	7.5 kPa varus	15 kPa varus	22.5 kPa varus	7.5 kPa valgus	15 kPa valgus	22.5 kPa valgus
Medial compartment							
Tibia							
Central subregion of MT	2.02	1.97 (− 2.5)	1.89 (− 6.4)	1.88 (− 6.9)	1.95 (− 3.5)	2.00 (− 1.0)	2.03 (0.5)
External subregion of MT	1.27	1.25 (− 1.6)	1.21 (− 4.7)	1.22 (− 3.9)	1.26 (− 0.8)	1.25 (− 1.6)	1.27 (0)
Internal subregion of MT	1.55	1.48 (− 4.5)	1.42 (− 8.4)	1.42 (− 8.4)	1.57 (1.3)	1.55 (0)	1.59 (2.6)
Anterior subregion of MT	1.45	1.36 (− 6.2)	1.36 (− 6.2)	1.38 (− 4.8)	1.37 (− 5.5)	1.43 (− 1.4)	1.35 (− 6.9)
Posterior subregion of MT	1.29	1.26 (− 2.3)	1.25 (− 3.1)	1.21 (− 6.2)	1.28 (− 0.8)	1.27 (− 1.6)	1.38 (7.0)
Femur							
Central subregion of cMF	2.12	2.03 (− 4.2)	1.94 (− 8.5)	1.94 (− 8.5)	2.16 (1.9)	2.06 (− 2.8)	2.13 (0.5)
External subregion of cMF	1.58	1.50 (− 5.1)	1.45 (− 8.2)	1.43 (− 9.5)	1.58 (0)	1.51 (− 4.4)	1.57 (− 0.6)
Internal subregion of cMF	1.52	1.41 (− 7.2)	1.34 (− 11.8)	1.34 (− 11.8)	1.53 (0.7)	1.48 (− 2.6)	1.51 (− 0.7)
Lateral compartment							
Tibia							
Central subregion of LT	2.68	2.59 (− 3.4)	2.55 (− 4.9)	2.63 (− 1.9)	2.51 (− 6.3)	2.47 (− 7.8)	2.44 (− 9.0)
External subregion of LT	1.41	1.29 (− 8.5)	1.29 (− 8.5)	1.29 (− 8.5)	1.38 (− 2.1)	1.33 (− 5.7)	1.35 (− 4.3)
Internal subregion of LT	1.95	1.87 (− 4.1)	1.90 (− 2.6)	1.95 (0)	1.84 (− 5.6)	1.82 (− 6.7)	1.78 (− 8.7)
Anterior subregion of LT	1.51	1.52 (0.8)	1.51 (0)	1.51 (0)	1.46 (− 3.3)	1.42 (− 6.0)	1.45 (− 4.0)
Posterior subregion of LT	1.38	1.36 (− 1.4)	1.31 (− 5.1)	1.30 (− 5.8)	1.31 (− 5.1)	1.28 (− 7.2)	1.30 (− 5.8)
Femur							
Central subregion of cLF	1.97	2.05 (4.1)	2.08 (5.6)	2.07 (5.1)	2.04 (3.6)	1.91 (− 3.0)	1.96 (− 0.5)
External subregion of cLF	1.38	1.50 (8.7)	1.49 (8.0)	1.49 (8.0)	1.40 (1.4)	1.31 (− 5.1)	1.29 (− 6.5)
Internal subregion of cLF	1.59	1.72 (8.2)	1.69 (6.3)	1.68 (5.7)	1.67 (5.0)	1.57 (− 1.3)	1.64 (3.1)

Cartilage thickness was quantified in a human cadaveric knee joint subjected to increasing varus–valgus loading. Data are given as mean thickness (mm) [percentage change versus unloaded configuration (%)]. See Table 2 for additional details

*MT* medial tibia, *cMF* central medial femur, *LT* lateral tibia, *cLF* central lateral femur

**Fig. 5:**
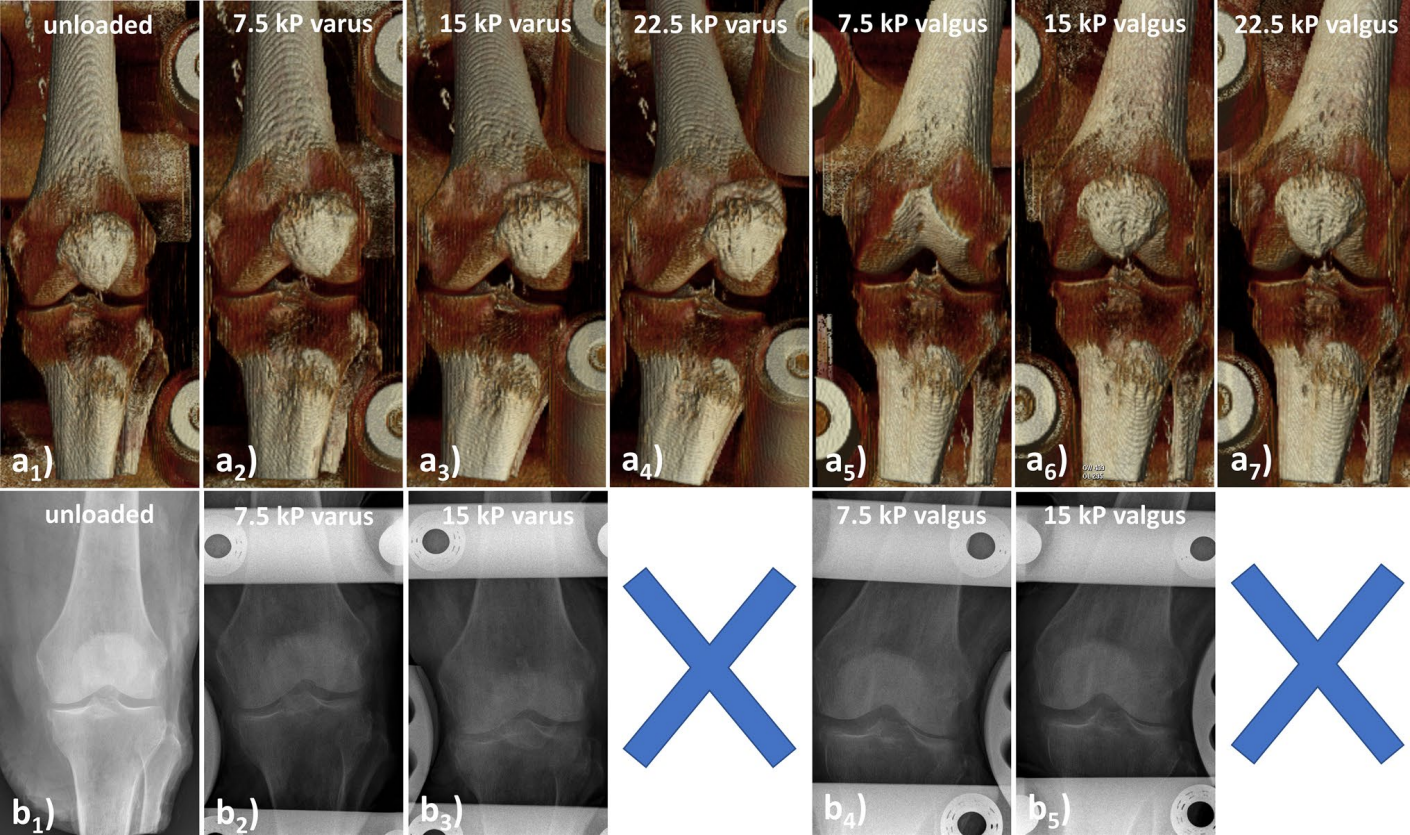
CT volume renderings (**a**) and radiographs (**b**) of the human cadaveric knee joint subjected to varus and valgus loading. Shaded volume renderings (**a**) were obtained in the unloaded configuration (**a**_**1**_), at 7.5 kPa varus (**a**_**2**_), 15 kPa varus (**a**_**3**_), 22.5 kPa varus (**a**_**4**_), 7.5 kPa valgus (**a**_**5**_), 15 kPa valgus (**a**_**6**_), and 22.5 kPa valgus (**a**_**7**_). Because of variable flexion and rotation, the volume-of-interest was centered on the knee as well as rotated and angulated to allow for unobstructed visualization of the joint space. Due to force restrictions of the stress radiography device anteroposterior radiographs could not be obtained at 22.5 kPa (indicated by blue crosses), but only in the unloaded configuration (**b**_**1**_), at 7.5 kPa varus (**b**_**2**_), 15 kPa varus (**b**_**3**_), 7.5 kPa valgus (**b**_**4**_), and 15 kPa valgus (**b**_**5**_)

For CT studies, qualitative and quantitative evaluation confirmed the findings above. Figure 5a gives detailed volume renderings in the different loading configurations. In response to varus loading, mean joint space widths (JSWs) of the MFTC decreased from 4.6 ± 0.6 mm (*δ*_0_) to 4.0 ± 0.6 mm (*δ*_var1_), 3.5 ± 0.8 mm (*δ*_var2_), and 3.4 ± 0.6 mm (*δ*_var3_). Correspondingly, in response to valgus loading, mean JSWs of the LFTC decreased from 7.2 ± 1.5 mm (*δ*_0_) to 5.2 ± 0.6 mm (*δ*_vlg1_), 4.8 ± 0.6 mm (*δ*_vlg2_), and 4.4 ± 0.9 mm (*δ*_vlg3_). For the unloaded joint compartments, i.e. the LFTC under varus loading and the MFTC under valgus loading, gradually distended JSWs were found, e.g. LFTC under varus loading: 7.2 ± 1.5 mm (*δ*_0_), 9.1 ± 2.0 mm (*δ*_var1_), 10.5 ± 0.9 mm (*δ*_var2_), 11.5 ± 0.3 mm (*δ*_var3_); mean JSWs of the medial femorotibial compartment under valgus loading: 4.6 ± 0.6 mm (*δ*_0_), 7.3 ± 2.1 mm (*δ*_vlg1_), 8.3 ± 2.6 mm (*δ*_vlg2_), 8.7 ± 2.7 mm (*δ*_vlg3_).

For radiography, similar observations were made (Fig. 5b). Mean JSWs of the MFTC decreased from 4.5 ± 0.8 mm (*δ*_0_) to 3.7 ± 1.0 mm (*δ*_var1_), and 3.0 ± 0.6 mm (*δ*_var2_) in response to varus loading. Correspondingly, mean JSWs of the LFTC decreased from 6.5 ± 0.8 mm (*δ*_0_) to 5.1 ± 0.1 mm (*δ*_vlg1_), and 4.6 ± 0.2 mm (*δ*_vlg2_) in response to valgus loading.

Flexion of the knee joint gradually increased in response to loading, from 20° (*δ*_0_) to 29° (*δ*_var1_), 45° (*δ*_var2_), and 48° (*δ*_var3_) as well as to 23° (*δ*_vlg1_), 25° (*δ*_vlg2_), and 28° (*δ*_vlg3_). Similarly, rotation of the knee joint gradually increased, too, from 13° external rotation (*δ*_0_) to 17° (*δ*_var1_), 25° (*δ*_var2_), and 29° (*δ*_var3_), while it remained unchanged in response to increasing valgus loading.

**Fig. 6:**
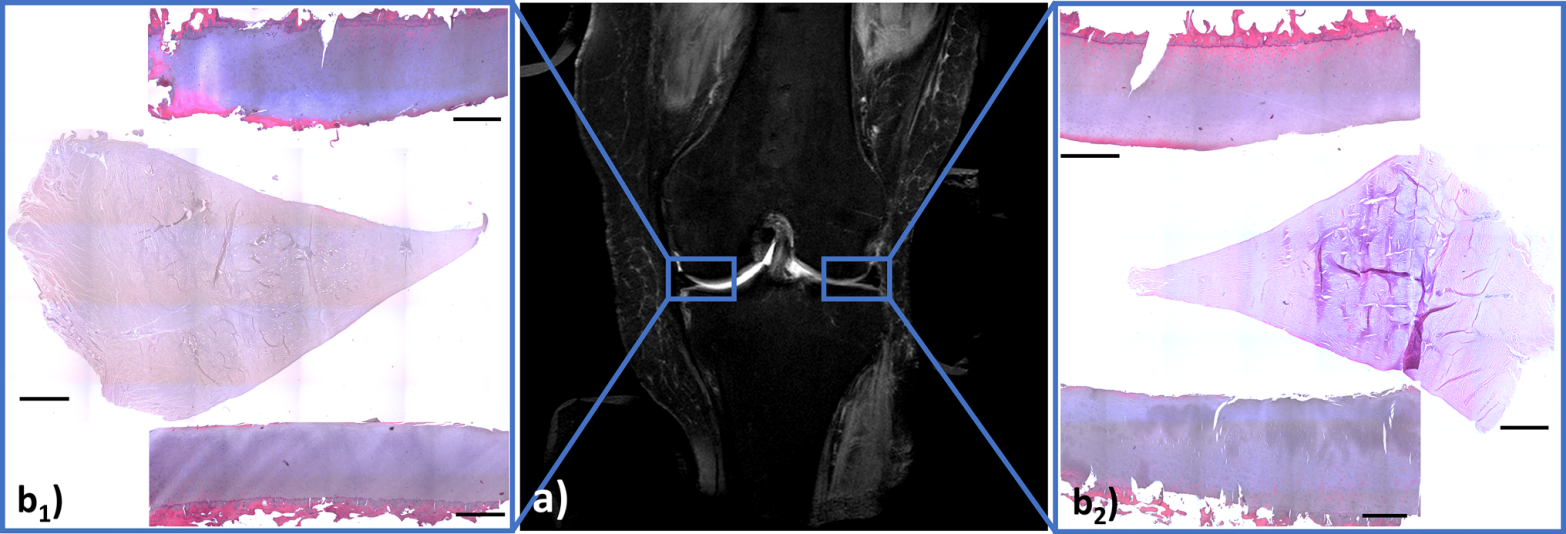
Synopsis of histological reference analysis. Coronal reference MR image [PDw-fs (**a**)] of the human cadaveric knee joint in the unloaded configuration. Light blue boxes indicate the site of cartilage and meniscus harvesting for subsequent histological analysis of the medial (**b**_**1**_) and lateral (**b**_**2**_) femorotibial joint compartments. Sampled femoral and tibial cartilage regions are shown at the top and bottom of the boxes, while the medial and lateral meniscus samples are positioned in-between. Cartilage samples were grossly intact with slight signs of early degeneration, i.e. focal surface disintegration, hypercellularity, and superficial proteoglycan de-staining. Medial femoral cartilage, however, displayed moderate-to-severe signs of degeneration, i.e. cellular cloning, clefting, and incipient tissue loss. Similarly, the lateral meniscus displayed severe signs of degeneration such as fraying of the apex, focal matrix disorganization, fibrocartilaginous separation, and strong proteoglycan staining, while the medial meniscus was structurally intact. Hematoxylin–eosin staining. Bars in histological sections are scaled to 1 mm. Note that the light blue boxes are not to scale

Biomechanical reference evaluation of the sampled cartilage areas revealed considerable variability across the knee joint. Instantaneous Young’s modulus was quantified as 1.11 MPa (MF), 0.34 MPa (LF), 1.88 MPa (MT), and 1.92 MPa (LT). Figure 6 gives detailed results of the histological reference evaluation.

## Discussion

The most important findings of the present study are that (1) VVL of the human knee joint induces efficient compartmental compression, thereby providing an alternative loading mechanism for functional joint assessment and that (2) intra-tissue adaptations in response to VVL were reliably detected using high-resolution morphological MRI techniques.

Based on the developed MRI-compatible VVL device, pressure may be actuated along the joint line in exactly defined varus or valgus configurations as controlled by pressure. The relation of set pressure levels and resultant forces generated at the padded actuator was close-to-perfectly linear. Most likely because of unavoidable friction and leakage within the pressurized infrastructure, i.e. the pressure lines and the pneumatic mechanism, this association was not perfectly linear. Longitudinal force measurements confirmed that the pneumatic mechanism maintains constant forces over extended time periods, i.e. at least 30 min, which is a prerequisite for combining loading with advanced MR imaging techniques that are time-consuming and necessitate constant loading conditions to reduce movement artefacts. Even though reference pressure mappings indicated effective pressurization of the MFTC and LFTC, VVL induced considerably lower intraarticular pressures than compressive axial loading along the joint’s mechanical axis with ≥ 50 % body weight (thoroughly reviewed in [6]). Of course, forces in response to VVL were substantially lower because of the principally different loading mechanisms. Mechanistically, when applying loading along the joint line while keeping both the distal femur and proximal tibia fixed, the knee joint centre becomes the fulcrum (i.e. centre of rotation) in the presence of such bending moments. Naturally, this induces inherently different distributions of pressure across the joint. Unfortunately, literature data on the biomechanics of VVL in general and on intraarticular pressurization in particular are scarce. While a solid body of scientific evidence demonstrated that forces are increased in the MFTC in varus (mal-)aligned knees and in the LFTC in valgus (mal-)aligned knees [28, 36–39], direct in vivo measurements of stress and strain within the knee joint are challenging, which may explain the paucity of available data. Current knowledge is primarily based on human cadaveric knee joint studies, e.g. [28], instrumented implants during or after total knee arthroplasty (TKA) [40–42], or computational models [37, 43]. When axially loading human cadaveric knee joints at 1000 N and different loading axes, Agneskirchner et al. found strong correlations between the degree of varus or valgus and the distribution of mean and peak contact pressures at the MFTC and LFTC [28]. Even though the topographic pressure distribution was not systematically investigated in their study, the central and inner compartmental joint surface areas were most pressurized, while the peripheral areas experienced less stress and strain. These observations were in line with earlier studies that assessed the pressure distribution in the knee during TKA and found primary involvement of the inner and central areas of the MFTC and LFTC in VVL [40, 44]. Our morphometric cartilage measures confirmed this spatial heterogeneity in pressurization across the knee joint in response to VVL. Loading-induced decreases in VC and ThC are indicative of effective pressurization of the MFTC in varus (and of the LFTC in valgus) and even though the compartments were loaded in their entirety, changes in VC and ThC were most pronounced in the central and inner subregions, both for the femur and the tibia, which confirms these earlier in vitro and in vivo data. In our study, further subregional analysis revealed that decreases in ThC were—by trend—larger in the posterior than anterior subregions of the tibia, especially at higher loading intensities. This finding may be explained by the concurrently increasing joint flexion angles as a result of loading, which not only decrease the contact areas and shift them in the posterior direction [45], but also increase the magnitude of cartilage contact deformation [46]. Discrepancies in study protocols, research questions and methodology (including loading regimes, measurement setups and overall framework conditions) as well as anatomy limit comparability of these findings to the conventional biomechanical literature.

In terms of cartilage deformation, our findings are well in line with previous stress MRI studies. Subjecting a healthy volunteer’s knee joint to a compressive axial load of 50% body weight, Subburaj et al. determined decreases in ThC of 0.074 mm (MFC), 0.003 mm (LFC), 0.071 mm (MT), and 0.061 mm (LT) based on mean (unloaded) ThC values of 1.42 mm, 1.49 mm, 1.35 mm and 1.71 mm, respectively [5]. Amounting to relative changes that ranged from − 1.0 to − 6.9%, the range of loading-induced changes reported in their study is comparable to our study. Interestingly, they also found cartilage deformation to be considerably larger in the MFTC than the LFTC, which was mainly due to the fact that the cartilage of the LF underwent hardly any changes. In contrast, loading induced largely consistent decreases in the cartilage thickness of the MF, MT and LT. In line with earlier in vivo studies reporting similar findings for axial loading [46, 47], our study, too, confirmed these findings. Consequently, effective cartilage pressurization when loaded along the joint axis (i.e. axially) and perpendicular to it (i.e. varus–valgus) seems similar and again confirms the mechanistic equivalence of VVL as compared to compressive axial loading, while keeping logistical and infrastructural demands manageable.

While stress radiography of the knee has been performed with variable loading intensities ranging from 3 to 30 kPa [21], our results indicate that load responsiveness of cartilage may be the greatest at loads of up to 15 kPa. Higher loading intensities (of up to 22 kPa) produced smaller and less consistent intra-tissue adaptations and may increase the risk of iatrogenic complications. It remains speculative why we could not find a linear relationship between loading intensity and relative changes in ThC for high-loading intensities. Possible explanations involve the more focal compartmental loading with more severe misalignment [28], possible force dissipation due to secondary adaptive flexion and external rotation, and increasingly relevant static stabilizers in unphysiological joint configurations [48]. For practical considerations, VVL should therefore be realized within the recommended range of 0–15 kPa [21]. MRI measurements and subsequent morphometric quantifications were performed in the order 1–7 as detailed above. Even though an equilibration period of 5 min was obeyed after each change in pressure level, we cannot exclude the possibility that the morphometric measures are also affected by the measurement order. Cartilage relaxation is related to loading intensity with longer relaxation (and ongoing intra-tissue adaptations) observed for higher-intensity loading [32, 33] and full ultrastructural recovery may necessitate extended relaxation periods of up to several hours [49, 50]. Hence, additional studies need to evaluate if (1) the equilibration period is justified by striking a sensible balance between additional magnet time and sufficient tissue relaxation and (2) the measurement order affects the morphometric measurements.

Our study and others [5, 46, 47] indicate that valgus loading-induced changes in the cartilage of the LT are undulating and inconsistent. Possible explanations involve knee joint-specific anatomic and biomechanical aspects. For once, due to its concave geometry, the articular surface of the MT affords higher congruity with the MF condyle, more extensive femorotibial articulation, and—consecutively—consistent cartilage straining and deformation [5, 45, 51]. In contrast, the articular surface of the LT is convex, which decreases the area of articulation [51], and may be the reason for the inconsistent subregional cartilage deformation patterns as loads are borne in a more focal than areal manner. For another, cartilage deformation is significantly larger in the MFTC than the LFTC at higher joint flexion angles, in particular between 30° and 60° (as in our study) [46].

Whenever absolute cartilage deformation is quantified, the degree of pre-existent degeneration needs to be considered as degenerative cartilage is more compliant [34, 52] which considerably affects loading-induced changes in morphometric measures. Earlier studies demonstrated substantially higher variability in loading-induced changes in OA versus non-OA [5]. Against this background, the definition of the physiological response to loading hinges on a sound reference standard that is certainly easier to realize in ex vivo studies. Consequently, we performed histological and conventional biomechanical referencing of the (previously loaded) sampled cartilage regions. Except for the MF that displayed moderate-to-severe signs of histological degeneration, sampled cartilage areas displayed only slight-to-moderate degeneration throughout the joint. Although the controversy of aging and its role in OA is ongoing [53], this is not surprising given the body donor’s age of 78 years. Surprisingly, the medial meniscus was histologically intact, while the lateral meniscus displayed more severe degeneration. Even though the exact relation between cartilage and meniscus integrity (and pathology) remains speculative [54], not just in this very knee and patient, the decisive role of the meniscus in compartmental load transmission and its failure in OA warrants additional investigations for which the present study provides a solid framework.

In our study, morphometric analysis was performed on the basis of a high-resolution WATSc sequence that is commonly applied for cartilage segmentation [25] and was obtained at an in-plane resolution of 0.35 mm/pixel. Such high-resolution imaging is a prerequisite to detect subtle regional cartilage changes with sufficient precision and accuracy and to use cartilage deformation in terms of changes in ThC and VC as imaging biomarkers of cartilage and joint functionality. While overall, unenhanced CT and radiography indicated similar changes within the joint, these modalities are characterized by distinct disadvantages such as radiation exposure, indirect visualization of soft tissues (unless performed as a direct arthrography), and the inability to assess their compositional, (ultra)structural and functional adaptations. These immanent disadvantages render these modalities ill-suited for functional tissue and joint assessment, in particular in view of the ever-more increasing availability of MRI and its distinct advantages such as non-invasiveness, superior soft tissue contrast, and absence of ionizing radiation.

Our study has a number of limitations. First and foremost, we included only one human knee joint specimen. However, the present study’s primary objective was to demonstrate proof of concept for joint functionality assessment based on the validated pressure-controlled VVL device. Of course, future studies investigating larger specimen sizes are necessary to corroborate our findings and to define the physiological response to loading across the joint and its pathological aberrations. Second, we only assessed knee joint functionality within the framework provided by the static stabilizers, i.e. capsule, menisci, and cruciate and collateral ligaments, while the dynamic stabilizers, i.e. muscles, could not be assessed. Hence, when it comes to the in vivo translation of our findings, studies including healthy (non-OA) individuals and OA patients are required to bridge this translational gap. Similarly, once regulatory and medicolegal issues have been solved, this proof-of-concept study has to be followed by a comprehensive feasibility study that includes sufficient numbers of volunteers (without OA or knee pain) and patients (with OA and/or knee pain). Aspects related to the concept’s in vivo translation such as patient comfort, joint positioning, stabilization, and fixation, device safety and handleability, convenience of operation as well as measurement validity and reproducibility will then be addressed with the device in clinical operation. Future efforts need to be directed at keeping examination times as short as possible, possibly by including abbreviated MRI protocols [55], and loading as low as possible by balancing (still effective) loading intensity against (functionally meaningful) joint and tissue changes. In particular, switching the direction of loading, e.g. from varus to valgus in the same knee, necessarily involves repositioning of the joint, coils and/or principal device components, which is not only impractical, but also means additional examination time. Third, when loaded, the knee joint underwent substantial flexion and—to a lesser extent—rotation, in particular in the varus configuration, which is due to the non-constrained loading setup. For the sake of standardization of loading, joint motion in response to loading needs to be better controlled, for example by using more confining mounting supports. Yet, any additional mechanical confinement needs to be balanced against the loading-induced adjustments and adaptations of the joint. Fourth, we only assessed morphometric measures such as ThC and VC, while we did apply more advanced MRI techniques such as compositional sequences. By application of T2 or T1ρ mapping, further inferences on cartilage (ultra)structure and composition (beyond mere morphology) and their changes in response to loading may be made. Fifth, biomechanical reference evaluation using pressure-sensitive sensors may only provide rough estimates of actual pressurization as this evaluation was performed after arthrotomy, preparation, and partial transection, thereby altering the joint’s overall soft tissue balance, mechano-functional properties, and effectual load transmission.

In conclusion, the MRI-compatible pressure-controlled VVL device provides a new and innovative way of assessing knee joint functionality based on pressurization of the MFTC and LFTC. In this proof-of-concept study, effective compartmental pressurization is reflected by distinct loading-induced patterns of subregional cartilage deformation and indicates the methodology’s potential application in basic, translational and clinical research questions. Once substantiated by more refined imaging, biomechanical, and compositional cross-references, this approach may improve handleability, decrease logistical demands, and provide a safe and powerful alternative technique for stress MRI in the context of functional joint and tissue assessment.

## Electronic supplementary material

Below is the link to the electronic supplementary material.

Supplementary file1 (DOCX 25 kb)
