# Correction to: An MRI‑compatible varus–valgus loading device for whole‑knee joint functionality assessment based on compartmental compression: a proof‑of‑concept study

**DOI:** 10.1007/s10334-021-00944-x

**Published:** 2021-07-23

**Authors:** Oliver Said, Justus Schock, Nils Krämer, Johannes Thüring, Lea Hitpass, Philipp Schad, Christiane Kuhl, Daniel Abrar, Daniel Truhn, Sven Nebelung

**Affiliations:** 1 Department of Diagnostic and Interventional Radiology, Aachen University Hospital, Aachen, Germany; 2 Department of Diagnostic and Interventional Radiology, Medical Faculty, University Hospital Düsseldorf, University Dusseldorf, Moorenstraße 5, 40225 Düsseldorf, Germany; 3 Institute of Computer Vision and Imaging, RWTH University Aachen, Aachen, Germany

## Correction to: Magnetic Resonance Materials in Physics, Biology and Medicine (2020) 33:839–854 10.1007/s10334-020-00844-6

The article An MRI‑compatible varus–valgus loading device for whole‑knee joint functionality assessment based on compartmental compression: a proof‑of‑concept study, written by Oliver Said, Justus Schock, Nils Krämer, Johannes Thüring, Lea Hitpass, Philipp Schad, Christiane Kuhl, Daniel Abrar, Daniel Truhn and Sven Nebelung, was originally published Online First without Open Access. After publication in volume 33, issue 6, page 839–854 the author decided to opt for Open Choice and to make the article an Open Access publication. Therefore, the copyright of the article has been changed to © The Author(s) 2021 and this article is licensed under a Creative Commons Attribution 4.0 International License, which permits use, sharing, adaptation, distribution and reproduction in any medium or format, as long as you give appropriate credit to the original author(s) and the source, provide a link to the Creative Commons licence, and indicate if changes were made. The images or other third party material in this article are included in the article’s Creative Commons licence, unless indicated otherwise in a credit line to the material. If material is not included in the article’s Creative Commons licence and your intended use is not permitted by statutory regulation or exceeds the permitted use, you will need to obtain permission directly from the copyright holder. To view a copy of this licence, visit http://creativecommons.org/licenses/by/4.0/.

Open Access funding enabled and organized by Projekt DEAL.

The original article has been corrected.

