# Supplementary material for: An MRI-compatible varus–valgus loading device for whole-knee joint functionality assessment based on compartmental compression: a proof-of-concept study
Source: MAGMA. 2020 Apr 20;33(6):839–54. doi: 10.1007/s10334-020-00844-6 (PMC8302563; doi:10.1007/s10334-020-00844-6)
Supplement: Supplementary file 1 — Supplementary file1 (DOCX 25 kb) [file 10334_2020_844_MOESM1_ESM.docx]

**Supplementary File 1**

**Title:** An MRI-Compatible Varus-Valgus Loading Device for Whole-Knee Joint Functionality Assessment Based on Compartmental Compression: A Proof-of-Concept Study

**Manuscript Number:** MRMP-D-20-00016

**Authors**: Oliver Said, Justus Schock, Nils Krämer, Johannes Thüring, Lea Hitpass, Philipp Schad, Christiane Kuhl, Daniel Abrar, Daniel Truhn, Sven Nebelung

**Journal:** Magnetic Resonance Materials in Physics, Biology and Medicine

**Corresponding Author:** Sven Nebelung, MD, PhD, University Dusseldorf, Medical Faculty, Department of Diagnostic and Interventional Radiology, Dusseldorf, Germany; Sven.Nebelung@med.uni-duesseldorf.de

**Detailed Description of Histological and Biomechanical Reference Evaluation**

**Histological Reference Evaluation**

Following imaging studies and force validation, the central weightbearing femoral and tibial joint surfaces were identified and the coronal reference plane was defined by creating corresponding peripheral notches at the distal femoral condyles and proximal tibial plateau using a rongeur. After dissection of the collateral and cruciate ligament complex to gain full access to the joint, the adjacent joint surfaces were excised by means of an electric saw. Correspondingly, medial and lateral meniscus samples were harvested from the central body region by means of a scalpel. Here, the coronal reference plane was defined from meniscus apex to base using tissue-marking dye (Polysciences, Warrington, US). Subsequently, both the excised cartilage-bone material and the meniscus samples were sectioned along the coronal reference plane so that histological sections were prepared from the central and peripheral joint areas along this reference. Histological preparations and analysis were performed according to standard routines as published before [1-4]. Briefly, cartilage-bone samples were simultaneously decalcified and fixed using Ossa fixona (Diagonal, Münster, Germany), while meniscus samples were fixed in 4% paraformaldehyde. Then, all samples were embedded in paraffin, cut to 5-µm sections, stained with hematoxylin/eosin and Safranin O in line with standard protocols and imaged using a state-of-the-art microscope (digital light microscope Leica DMI6000 B, Leica, Wetzlar, Germany). To visualize cartilage and meniscus samples in their entirety, individual micrographs were merged into one image per sample using in-built software (Leica Application Suite X, Leica).

**Biomechanical Reference Evaluation**

Cartilage-bone material adjacent to joint surface areas sampled for histology was prepared for biomechanical reference characterization as before [5]. First, we cut cylindrical chondral samples from the material using a skin biopsy punch of 8 mm diameter (pfm-medical, Cologne, Germany). Second, we removed the subchondral bone using a standard scalpel, while we took meticulous care to obtain samples of uniform thickness. Third, we determined sample thickness using a standard digital micrometer (Mitutoyo 293-521; Mitutoyo, Tokyo, Japan). Fourth, we subjected the samples to unconfined compression tests on a universal mechanical testing machine equipped with a compressive piston (diameter: 20 mm) and a load cell (force range: 200 N) (Zwick/Roell Z2.5; Zwick/Roell, Ulm, Germany). More specifically, we compressed samples to a maximum strain of 21 % at a constant displacement rate of 0.15 % strain/sec. As the tissue’s response to loading is dependent on the complex interplay of fluid pressurization and fibril reinforcement, this strain rate was chosen to assess the former’s contribution over the latter [6]. TestXpert software (Zwick/Roell) was used to simultaneously record displacement and load data. Fifth, Instantaneous Young modulus was determined as the ratio of stress and strain by fitting a tangent to the strain range of 10% – 20%. Of note, samples were kept hydrated throughout the measurements.

**Bibliography**

1. Nebelung S, Post M, Knobe M, Tingart M, Emans P, Thuring J, et al. Detection of Early-Stage Degeneration in Human Articular Cartilage by Multiparametric MR Imaging Mapping of Tissue Functionality. Sci Rep 2019; 9: 5895.

2. Truhn D, Sondern B, Oehrl S, Tingart M, Knobe M, Merhof D, et al. Differentiation of human cartilage degeneration by functional MRI mapping-an ex vivo study. Eur Radiol 2019.

3. Nebelung S, Post M, Knobe M, Shah D, Schleich C, Hitpass L, et al. Human articular cartilage mechanosensitivity is related to histological degeneration - a functional MRI study. Osteoarthritis Cartilage 2019; 27: 1711-1720.

4. Nebelung S, Sondern B, Jahr H, Tingart M, Knobe M, Thuring J, et al. Non-invasive T1rho mapping of the human cartilage response to loading and unloading. Osteoarthritis Cartilage 2018; 26: 236-244.

5. Nebelung S, Sondern B, Oehrl S, Tingart M, Rath B, Pufe T, et al. Functional MR Imaging Mapping of Human Articular Cartilage Response to Loading. Radiology 2017; 282: 464-474.

6. Li LP, Herzog W. Strain-rate dependence of cartilage stiffness in unconfined compression: the role of fibril reinforcement versus tissue volume change in fluid pressurization. J Biomech 2004; 37: 375-382.
